# Myth of objectivity and the origin of symbols

**DOI:** 10.3389/fsoc.2023.1269621

**Published:** 2023-10-09

**Authors:** Shagor Rahman

**Affiliations:** Independent Researcher, Westfield, NJ, United States

**Keywords:** free energy principle, semiotics, epistemology, anthropology, economics, morality, social ontology, linguistics

## Abstract

An age-old challenge in epistemology and moral philosophy is whether objectivity exists independent of subjective perspective. Alfred North Whitehead labeled it a “fallacy of misplaced concreteness”; after all, knowledge is represented elusively in symbols. I employ the free energy principle (FEP) to argue that the belief in moral objectivity, although perhaps fallacious, amounts to an ancient and universal human myth that is essential for our symbolic capacity. To perceive any object in a world of non-diminishing (perhaps irreducible) uncertainty, according to the FEP, its constituent parts must display common probabilistic tendencies, known as statistical beliefs, prior to its interpretation, or active inference, as a stable entity. Behavioral bias, subjective emotions, and social norms scale the scope of identity by coalescing agents with otherwise disparate goals and aligning their perspectives into a coherent structure. I argue that by declaring belief in norms as objective, e.g., expressing that a particular theft or infidelity was *generally* wrong, our ancestors psychologically constructed a type of identity bound only by shared faith in a perspective that technically *transcended* individual subjectivity. Signaling explicit belief in what were previously non-symbolic norms, as seen in many non-human animals, simulates a top-down point of view of our social interactions and thereby constructs our cultural niche and symbolic capacity. I demonstrate that, largely by contrasting with overly reductive analytical models that assume individual rational pursuit of extrinsic rewards, shared belief in moral conceptions, i.e., what amounts to a religious faith, remains a motivational cornerstone of our language, economic and civic institutions, stories, and psychology. Finally, I hypothesize that our bias for familiar accents (shibboleth), plausibly represents the phylogenetic and ontogenetic contextual origins of our impulse to minimize social surprise by declaring belief in the myth of objectivity.

## 1. Introduction

I MET a seer,

Passing the hues and objects of the world,

The fields of art and learning, pleasure, sense,

To glean eidólons.

Ever the mutable,

Ever materials, changing, crumbling, re-cohering,

Ever the ateliers, the factories divine,

Issuing eidólons.

Exaltè, rapt, ecstatic,

The visible but their womb of birth,

Of orbic tendencies to shape and shape and shape,

The mighty earth-eidólon.

The noiseless myriads,

The infinite oceans where the rivers empty,

The separate countless free identities, like eyesight,

The true realities, eidólons.

- Excerpts from Walt Whitman's poem *Eidólons*

The boundary between us and non-human animals is likely symbolic.^1^ Though semiotics and information processing are a fundamental phenomenon throughout biology and physics (Deacon, 1998; Fields and Levin, 2020), we are the one species that employs symbolic thought and expression to cognitively displace time and space and inhabit a social reality (Searle, 2010a). We pray to icons, worship written texts, watch stories, don uniforms, purchase branded goods, salute flags, and gift material alms to our dead. Our own intimate sense of identity is a psychological conception constructed within extensive cultural units (Leflot et al., 2010).

The question is *what are the developmental and historical foundations of this capacity? What motivates us to acquire the complex cognitive architecture to learn and adopt social conventions, language, and symbolic ornamentation?* Rethinking the set of core factors that generally govern social science models, that often assume individual rational pursuit of extrinsic rewards, may allow for better modeling in the deeply complex interactions we face, economically, psychologically, and linguistically. I believe any answer to these questions involves an appreciation for the dynamic nature of identity, i.e., how disparate lower-level constituents join a coherent hierarchical and self-organizing structure. Adopting Levin (2019) notion that the scope of cognition and agency that constitutes a *self* is analogous to the projected illumination of a *light cone* (p. 1):

I propose a fundamental definition of an Individual based on the ability to pursue goals at an appropriate level of scale and organization[…]. Any Self is demarcated by a computational surface—the spatio-temporal boundary of events that it can measure, model, and try to affect. This surface sets a functional boundary - a cognitive “light cone” which defines the scale and limits of its cognition.

Meeting Levin's metaphor, my argument is that our symbolic capacity is founded on the ability to take, or imagine, a top-down perspective and shine the light cone upon our social interactions and thereby scale the scope of our capacity through a cultural niche. Moral objectivity, of course, requires taking a *neutral*, third-party view, i.e., *above the fray* of individual perspectives. As we will explore in more detail later, Judith Burkart and colleagues have pointed out that our use of objective, third-party perspective distinguishes social norms exhibited by primates and human morality (Burkart et al., 2018). This is broadly true of all use of all symbols and the social reality we inhabit. Words have meaning as speakers conform to deontic conventions to maintain abstract meaning across individual subjective contexts though, as we will explore (see Section 3.1), objectivity remains an elusive construct. Despite the immutability of subjectivity, my use of the phrase *myth of objectivity* seeks to capture an often earnest and often conspicuous attempt to adopt a neutral *bird's eye* perspective upon social relationships is fundamental to our use of symbols.

By myth, I aim to emphasize that humanity's symbolic innovation was less a discovery of an underlying structure and more a social construction that served group interest and individual cognitive *sensibility*, i.e., it minimized surprises related to social interactions and facilitated shared expectations. As we will explore, although behavioral and social sciences prefer to describe behavior and motivation in terms of a rational pursuit of individual goals, we continually defy, or demonstrate analytically, a need for such defiance of these expectations by *identifying* with others, often using arbitrary markings and particularly accents (Section 4). However, by indicating our central symbolic myth is objectivity and not a supernatural entity, I emphasize that our symbolic consciousness is inherently an earnest search for truth based on an attempt to transcend individual subjectivity and form an objective viewpoint and that this *technical transcendence* emerged to solve traditional problems of a meek primate during the Pleistocene and, specifically, to update our beliefs about the beliefs of our conspecifics, expanding the scope of our cultural niche (Section 5). This is exemplified in the quintessentially moral and foundational concept of our economic system known as *social commons*, i.e., those resources and elements of production that extend beyond private ownership and are often facilitated by a top-down perspective of our economic and civic institutions. These and similar factors became understood specifically in light of the extremely reductive neoclassical economic model (Section 3.2). An equivalent moral conception anchors the otherwise arbitrary signification process of our linguistic systems, i.e., *transcendental signification*, which emerged by contrasting with the equally reductive structuralists (Section 3.1). Finally, our moral practice itself is an extension of emotional mental states, a phenomenon that is now studied in species that are as diverse as people and fruit flies by contrasting behavioral expectations of behaviorists (Section 3.3).

Symbolic thought can be viewed, in accordance with the free energy principle (FEP), as a culmination of a broader epistemic ontology. Rather than beginning its analysis with stable systems or well-defined agents in pursuit of selfish goals (Section 3.2), the FEP emphasizes the imperative for subjects to establish and update beliefs about relevant features of its environment, beginning with the partition that distinguishes and defines its own identity. Life, in other words, is inherently scientific in the sense that living systems seek evidence for internal models of their lived world. We will see later that this can viewed as seeking evidence for generative models of how our sensations are caused (see Section 2).

This evidence-seeking behavior may be an existential imperative. Under the free energy principle, this is neatly summarized as self evidencing (Hohwy, 2016), wherein the primal inquiry of the Self develops and evolves through an implicit version of Hofstadter and Sander (2013) view of symbolic thought as inherently analogical, i.e., just as we extrapolate our conceptual understanding by finding connections among otherwise distinct events or experiences, biological entities are connected through the interpretation of similarities. In FEP terms, this is a common prior belief that forms a coherent, if fuzzy (probabilistic), Self. Symbols are a way of explicitly engaging with this existential process of self and environmental understanding. Viewed in another way, science itself is an explicated and shared version of the epistemic nature of existence grounded in a religious orientation, primarily one that is geared toward signaling our own and understanding the beliefs of our conspecifics.

Although the FEP uses *belief* specifically as defined in Bayesian statistics,^2^ i.e., the movement of particles in an atom and the behavior of organelles in a cell appear to be a statistical model of their environment, Thomas Bayes originally conceived his insights into probability within a religious context (Earman and The Society of Christian Philosophers, 1993). As the FEP emphasizes the scale invariant properties of self-organization across cells (Friston, 2013), society (Albarracin et al., 2022), and culture (Ramstead et al., 2016; Constant et al., 2019), it is in the originally Bayesian context of religion, e.g., belief in supernatural entities and imaginary cultural narratives that represent a unique ability to *explicitly* compute or conceptualize foundational elements of personal identity and give shape to our social reality, that I attempt to extend its scale.

The FEP's emphasis of statistical boundaries, i.e., Markov blankets (MB), as a foundational element in the computations of self-organizing and biological systems, offers a useful view of emotions, norms, societies, and the morphological form of identity that I apply to our social reality. Our impulse to inhabit symbolic selves is anchored primarily in morality, i.e., the shared belief in objective norms and moral character types based on responses to established behavioral expectations. Note that I am taking a pragmatic (although as with all claims around morality, not incontrovertible) view of morality, as a symbolic abstraction of social norms and grounded in the expectation of shared faith in a cultural niche (Section 3.3). This takes an evolutionary viewpoint, as social norms observed in similar primates and other nonhuman animals suggest our ancestors inhabited a similar normative structure. This does not confine morality to this view but hopefully offers a convincing and useful starting point. Most importantly, it offers morality as a useful gradation to understand our developmental and historical ascent to the realm of symbolic thought.

The view of Markov blankets as fuzzy, mutable, and multiplicative allows us to understand how various distinct cognitive light cones are woven together to form more complex selves. This characterization was developed by Andy Clark who asserted, in defense of the extended mind thesis, that the MB should not be thought of as a singular and rigid structure (Clark, 2017). The attributes of fuzzy, mutable, and multiplicative need some careful qualification. By fuzzy, I refer here to the probabilistic nature of representations, even when symbolic or discrete. For example, I may believe that you are American but there is a nontrivial possibility that you may be Italian, and this is represented probabilistically in the biophysical configuration of my brain. There is also a sense in which Markov blankets can be fuzzy, in the sense that strict conditional independence can be replaced by weak conditional independence. With this relaxation, it can be shown that any dynamical system (with sparse coupling) of sufficient size admits multiple Markov blankets with probability one Sakthivadivel (2022). Crucially, there is no unique Markov blanket: there can be Markov blankets of Markov blankets and so on in a scale-free sense. Generally, smaller Markov blankets (e.g., cells in a body) exist for a shorter period of time than Markov blankets of Markov blankets (e.g., conspecifics in a population). This is what is implied by mutable and multiplicative.

This view of the Self as constitutive of a shared bias of sub-units informed by fuzzy, layered, and ultimately movable boundaries that contextualize a common sentient experience frames the subjective, qualitative hedonic valence that allows agents to distinguish between good and bad experiences in order to navigate its environment favorably and persist across generational lineages. Under the FEP, *good* and *bad* experiences are simply *predictable* and *surprising* experiences, respectively. This follows from the fact that navigating the sensed world entails a minimization of free energy—and free energy can be read as surprise. Crucially, negative free energy can also be read as the log evidence for the generative models upon which free energy rests. In other words, avoiding surprising sensations is maximizing the evidence for generative models of how those sensations were caused (please see Section 2).

Affect and emotion can be seen in this view as enabling complex organisms to respond to varying bodily and environmental demands by compartmentalizing various environmental needs and prioritizing among disparate internal systems, this amounts to nested Markov blankets enjoined into a coherent identity (Solms, 2019, 2022; Anderson, 2022), and sets the ground for in-group preferences and social identity (Moffett, 2013; Masuda and Fu, 2015; Palacios et al., 2020). Coherence of identity, however, does not imply unity (Levin, 2019), it is closer to a political coalition than the harmony of an infantry march.

Cooperation broadly emerges not just as expectations of future reciprocal selfish rewards but in the very ambiguities of the *self*. Organelles were once free-roaming molecules that were appropriated within a cellular identity, and cells in turn enjoined, partly by receiving information signals through gap junctions in their formerly rigid boundaries, to form parts of more complex organisms (Section 3.2). Daniel Dennett and Michael Levin specifically employ the mathematical framework from economics, the prisoner's dilemma, based on rational and selfish interested actors, to demonstrate how bioelectricity enables otherwise individuated cells to occupy multiple identities by sharing information through gap junctions using protein connection (Levin and Dennett, 2020). As Michael Levin put it (Carroll, 2021),

“We're doing […] simulations of prisoner's dilemma, where the agents, instead of just cooperating and defecting […] they can merge. […] you find out that cooperation doesn't just emerge, it's inevitable, because you can't cheat against yourself, because yourself is now bigger.”

Regarding societies, each chimp and lion is part of a community and pride of its individual subjective understanding. The boundaries of a society are actively inferred or interpreted by each potential group member based on individual intimacy (Moffett, 2019). In addition to the physical and social partitions that parallel our primate relatives, we inhabit communities, clans, countries, companies, and various “micro-selves” (Ramstead et al., 2016, p. 15), contextualizing our expectations and follow intricate norms that would otherwise be computationally intractable. As the basis of sentient life can be understood in FEP terms as a search for and affirmation of self identity that grounds the subsequent epistemic environmental search (Hohwy, 2016), human psychology can likewise be understood as a continual search for symbolic selves within a social reality.

As symbols can be seen as an explicit representation of implicit cognitive representation in other animals, the social rituals and belief systems of religion are simply a more elaborated explication of fundamental elements of our symbolic social reality, as I attempt to elucidate by contrasting with reductive, selfish views of biology and social sciences (see Section 3.2). I propose a causal narrative that maps our trajectory from a non-symbolic primate and *deconstruct* modern symbolic social systems to reveal their core components (Section 3.1). Regarding the latter, the structure of the social reality we each inhabit, including our personal self-conception (MacKinnon and Heise, 2010), is most *explicitly* evident in ancient and modern religious practices wherein, according to researchers Candace Alcorta and Richard Sosis, the expression of belief in arbitrary narratives involving supernatural entities are used both as an extrinsic signal of social identity and intrinsic psychological motivation in shaping personal identity. Religious belief fulfills a social and innate need to bind us to other social constituents, often through social rituals (Alcorta and Sosis, 2005). The enjoining of individual identity to a symbolic Self is represented in Jungian scholar M.L. Von Franz description of various mythological motifs collectively as the “cosmic man,” as he put (Jung et al., 1968, p. 274–277):

The Cosmic Man appears in many myths and religious teachings [as] a common representation of the Self in myths and dreams. […] He appears as Adam, as the Persian Gayomart, or as the Hindu Purusha […] In ancient Chinese […] a colossal divine man called P'an Ku. [T]he Cosmic Man is not only the beginning but also the final goal of all life—of the whole of creation. […] The whole inner psychic reality of each individual is ultimately oriented toward this archetypal symbol of the Self.

I characterize this feature of religion as concertizing the transcendent view of the Self as inclusive of an expansive and coherent cultural niche. Myth and social ritual provide a psychological motive to moral practices, yielding their extensive cooperative benefits (Curry, 2016), which is an elaboration of the minimal symbolizing of the Self as constituted by declaring explicit beliefs in moral objectivity. Religion serves to integrate each personal story within a unified cultural narrative. Moreover, archaeological findings such as the Rising Star cave in South Africa not only demonstrate symbolic thought through fossilized social ritual, they suggest the ancient role of belief in the supernatural served for individual and cultural identity (Berger et al., 2015).

The pattern of the symbolic Self is similarly explicated in modern and ancient storytelling. Chris Booker applied an equivalent Jungian perspective to cultural narratives from Gilgamesh to Terminator 2, finding that plot structures frequently involve a protagonist confronting a threat not just to his or her own well-being but to the greater culture or symbolic Self that ultimately determines their own fortunes and personal identity (Booker, 2004).

Religion and storytelling are a higher-order symbolic explication of the implied faith humanity possessed in an abstract symbolic Self and cultural identity, promoting the pursuit of synthetic intimacy of trusted conspecifics that demonstrate allegiance to its corpus. They also serve to establish a cultural *common ground* to affirm and construct social expectations by aligning subjective perspectives to a common reference frame. In my view, they explicate the technically transcendent, *from above* perspective of social interactions. This reinforces moral principles common to most cultures, e.g., though shall not kill (in-group members), and also expands it to broader conventional norms that have more flexibility as cultural and environmental challenges evolve. The questions I seek to answer are as follows: *Why are supernatural deities, heroes, and villains so central to the human experience? What does this imply for language and our symbolic capacity? Are beliefs in supernatural deities and villains that threaten a symbolic cultural identity central to our social reality?*

The bulk of the evidence I draw on to support this developmental and historical narrative is drawn from analysis that approaches social and even biological systems as if they were rigid structures and composed of selfish agents. This overly reductive scientific stance represents an important tendency of Western Educated Industrialized and Democratic (WEIRD) societies. It is worth understanding Henrich's proposed distinctions of WEIRD people. We are unique, according to Henrich (2020), in our strongly individualistic and analytically oriented perspectives, ideologies, and cultural institutions. This encouraged, in addition to the scientific and industrial revolutions, the stance to reduce behavioral motivation to extrinsic forces to the exclusion of subjective, intrinsic, and emotional internal states. Behaviorists in psychology, neoclassical economists, and structural linguistics all attempted to reduce the complexity of our social behavior to observable and *objective* causes (Section 3).

However, this viewpoint is not novel to modern Western culture. Extant nomadic hunter-gatherers or multi-band societies are similarly analytically oriented (Section 3.1). This suggests that the tendency that informs the scientific view of rigidly individuated selfish agents motivated exclusively by extrinsic rewards seen in economics (Kay and King, 2020), biology (Noble, 2017), and psychology (Anderson, 2022) represents a culmination of an ancient and human bias to construct hierarchical taxonomies of our world, conspecifics, and personal identities.

What have these reductive and selfish views concluded? Many researchers have most effectively used them as Null Hypotheses. The implications can be largely bucketed into three areas. Firstly, there is no underlying mechanism that allows individuated agents to fully *resolve* complexity and uncertainty and account for the structures we construct and inhabit. Secondly, the Self is not a rigid and immutable construct. Thirdly, humanity employs moral conceptions to harness the ambiguities of the Self, yielding the cooperative gain earned from integrating disparate agents. As hunter-gatherers have long inhabited arbitrarily demarcated social systems that rely on mythologized belief to foster mutual trust in their symbolic cultural identity and affirmed through various practices that encourage group alignment and labeling deviants, so too do our modern social systems and symbolic psychology.

Regarding economics (Section 3.2), in contrasts to the neoclassical economists' expectations of rational, self-interested agents with stable preferences (Friedman, 2008; Jehle and Reny, 2011), where group identity is generally treated as a psychological bias that impedes the rational pursuit of selfish utility (Kahneman, 2003; Simler and Hanson, 2018), the existence of firms, labor unions, educational institutions, and even countries occurs precisely to leverage the ability of economic agents to align their individual *utils*, interests, and preferences with others as part of a larger group identity. Most fundamentally, all economic systems rely on the concept of *social trust* in a web of inhabited groups (Simon, 1992; Fukuyama, 1996). Social trust and similar concepts such as *social commons* and *public goods* are classified as *market failures* and are effectively moral in nature, i.e., the necessary expectations that economic agents are reliable and do not sell goods that are harmful or generally misrepresent their quality. These Neoclassical market failures suggest a requisite shift from the subjective perspective of an individual selfish agent and toward a symbolically social, morally objective, or technically transcendent orientation.

Of course, our group biases are a double-edged sword: on the maladaptive side of our social systems, terrorist organizations and criminal gangs exists and are able to recruit members, according to Michael Hogg, because the risk they pose for their constituents is valued less than the assurance a group identity provides in times of social uncertainty (Hogg, 2000), and where social trust is inadequate. This is worth noting because it emphasizes, more so than firms and countries, that aligning our attention, preferences, interests, and ultimately our identity is not just a response to extrinsic threats and rewards but an innate psychological impulse.

In the field of linguistics Claude Lévi-Strauss and others known as *structuralists* attempted to reduce various languages and cultural myths to a stationary system, identifying the core components of this system identified across cultures and languages. However, this attempt was undermined by Jacques Derrida and Jacques Lacan who demonstrated that language unavoidably involves bespoken interpretations that are influenced by historical, social, cultural, and individual context (Section 3.1). The lack of fixed or common boundaries around signified concepts in linguistic systems however does not mean there are a few common elements. Linguistic signifiers (words) and concepts firstly gain meaning through binary opposition, wherein it is the distinction between words and their meaning that allows us to understand individual words, concepts, and expressions, e.g., cat vs. hat (signifier opposition), and black vs. white (signified opposition).

However this play of differences needs to exist as a predictable structure in order to be intelligible and functional across individuals within a given culture to enable complex symbolic communication, this is achieved by often arbitrarily privileged concepts, what Derrida called *transcendental signifieds*. For example, *God*, the monarch, democracy, liberty, etc. possess unquestioned value and serve as the top of a hierarchy below which other concepts are arranged. Even more prosaic concepts are given a normative quality that we can understand as giving language a common hierarchical structure within a given cultural context that orients the subsequent values or philosophical arguments, e.g., *presence* is privileged over *absence* that leads to, for example, Socrates' philosophical argument of speech over written text, an argument that effectively relies on a very arbitrary belief, akin to mythology (Derrida and Bass, 1998; Collins and Mayblin, 2011; Derrida and Spivak, 2016). This is similar to Heidegger's distinction of ontology vs. ontics and his claim that Western philosophy tends to concertize foundational ontological questions as ideas, e.g., Platonic forms, God, reason, etc. while failing to address the very nature of these or any beings. Both Derrida's transcendental signifiers and Heidegger's ontics critiques can be understood as the tendency to anchor the technical transcendent perspective to a fixed point.

The emergence of transcendental signifieds enable our symbolic consciousness to satisfy the FEP stated imperative of epistemically seeking general coherence, framing our linguistic systems as the product of a search for a symbolic Self suggests that privileging certain concepts as *good* for the *collective self* is necessary. The notion of coherence here is technically important. From the perspective of the FEP, an ensemble of creatures or conspecifics, constitutes a collection of Markov blankets, each trying to minimize their free energy or surprise by acting in a way that renders their co-constructed niche maximally predictable. The inevitable consequence of this is that the exchange among conspecifics tends to minimize the joint free energy through some kind of belief sharing and convergence on a shared generative model or narrative that can be read as *common ground*, i.e., a practice observed in the cooperative exchanges of children, where a shared identity motivates participant to establish sufficient trust and co-construct solutions to problems (Tomasello, 2016). Mathematically, this manifests as something called generalized synchrony or synchronization of chaos, namely, a coherent and mutually predictable exchange that constrains sensory trajectories to a synchronization manifold. This fundamental tendency to a non-equilibrium steady-state (with minimum free energy) is relatively straightforward to simulate and again foregrounds the emergence of common ground and a shared generative model (Friston and Frith, 2015; Isomura et al., 2019). Heuristically, if auditory sensations are generated by you or me, I can minimize my surprise by ensuring we are singing from the same hymn sheet, and all that needs to be inferred is whose turn it is to sing. However, to infer we share a generative model means I have to identify you as being something like me (i.e., we share a language or dialect). We will return to this key issue of identification and discrimination later.

Hence, although both Derrida's transcendental signification and Heideggar's ontics were presented biases that thwarted sound philosophical and political arguments, expressing beliefs in objective norms, even implicitly, whether in abstract social ideals or supernatural identities, effectively provides *evidence* of a shared symbolic Markov blanket and thus represents a necessary component of symbolic communication. It establishes a cultural common ground. It also aligns with the idea of “cooperative communication,” the idea that speakers are motivated to employ symbolic language to align mental states, i.e., beliefs, intentions, and emotions (Vasil et al., 2020), at least as an intermediate if not end objective (see Tison and Poirier, 2021). Declaring belief, whether in supernatural deities or principles, represents a fundamental deontic mode of social behavior that suggests a shared identity and subsequently motivates further alignment of mental states.

This ties to the last area that reductive analysis has proven useful as effectively a *Null-Hypothesis*. It also illuminates the contiguity of our symbolic practices with the animal kingdom because it applies to a broad range of organisms, i.e., emotions and norms (Section 3.3). Firstly, emotions represent a higher level of biological control seen in homeostatic autonomic processes, e.g., metabolism and thermoregulation, that drive compartmentalized systems toward stable set points. Researchers David Anderson and Ralph Adophs define emotions by contrasting the physiological responses of organisms that respond to things like predators and threats in ways that contrast with expectations of immediate stimulus response, forwarded by behaviorism, a school that sought to avoid the vexing problem of assuming subjective emotional states in non-symbolic animals. Instead, Anderson and Adolphs cast emotions as internal states that serve as “intervening variables,” motivating a range of behavior based on contextualized stimuli (Adolphs, 2013; Anderson, 2022).

Secondly, these emotionally motivated behaviors include the implicit social norms observed in cooperative animals including great apes, marmoset monkeys, dolphins, elephants, wolves, and certain rodents (Bekoff and Pierce, 2009; Burkart et al., 2018). Norms emerge from the inevitably variable interactions of social life given the individual and social challenges each agent confronts. The collective expression of anger by female chimps toward the alpha male (Boehm, 2012), or the convention of sticking one's butt in the air as a signal of submission in a within-group conflict, for instance, (Bekoff and Pierce, 2009), ensures stability of the social unit by exerting a harmonizing influence over the disparate needs of constituents and managing occasions of conflict and adversity while allowing for the benefits of bounded competition.

Norms thus enable social constituents to realize a more profitable position along what researchers Dupré and O'Malley call the “collaboration continuum,” (for an intuitive example of dynamics of the complex dynamics of cooperation and cooperation that this term implies, think of the cooperation within a sports team that exists simultaneously alongside individual team members striving to contribute the most, i.e., the MVP award (Dupré and O'Malley 2009, p. 1). Finally, emotions underwrite the social phenomenon of play where, for instance, primates and other mammals act out varying roles such as alpha and beta, shifting between the behavioral constraints and indulgences of these roles (Solms, 2022). These are the most important implicit phenomena we share with nonhuman animals that we explicit via symbolic expression.

The other main question I seek to explore is how we effectively *exported* these internal states as symbolic expressions, how it was that we transitioned from what Terrence Deacon labeled as the broader indexical and iconic signal processing seen across the animal kingdom, to our symbolic practices (Section 4). Given that our social systems evolve and persist in the face of potential free riders and deviants by minimizing social surprise with objective norms, moral concepts, and types, *how did we* (unlike other animals including our close primate kin) *develop the ability to do so?* The answer is likely found in some of the earliest signal processing we engage in and post utero, and how this enables us not only to socialize with intimates but ultimately move beyond them (Moffett, 2019; Kinzler, 2021).

Although the semiotician Jacques Lacan focused on our visual perception as the primal sensory modality when trying to understand the developmental of our symbolic Self conception, and focused on intimate caretakers as the most relevant context of psychological symbolic identification for a given subject, it is increasingly evident human infants instinctively synthesize intimacy using sounds, i.e., shibboleth (Liberman et al., 2017).^3^ We display a preference for our caretaker's accent in utero and later generalize this bias toward those with familiar accents (Kinzler, 2021).

While primates and other sufficiently complex and social animals rely on individual intimacy to cohabit with conspecifics in order to persist as a stable group (Moffett, 2013, 2019), we innately employ visual and audible markers to construct social categories over which we exhibit bias. Often investigated for its anti-social and harmful impacts, the instinctive bias to interpret accents to signal social categories, along with preferences over them, emerges in infancy (Kinzler et al., 2009; Kinzler, 2021). Though potentially harmful, this represents an ability to expand our social bonds by effectively synthesizing intimacy, a process that can be framed in semiotic terms as involving the transfer of cognitive sign meaning via analogy (Hofstadter and Sander, 2013), but an analogy based on a natural reference grounded in natural (i.e., indexical and iconic) representation; given that accents are fuzzy (i.e., probabilistic) yet reliable indicators of identity and norm abidance (McElreath et al., 2003).

This marks an important evolutionary and developmental step in our symbolically ordered world and cultural identity (Cohen, 2012; Moffett, 2019). The symbolic patterning of our psychology may not simply extend from our intimates to anonymous social relations, rather they are intertwined from early in our ontogeny via the interpretation of accents as dialectic. Although the depth of the psychological experience among our intimates might be most relevant individually, as a species it is the breadth of connections that are the most significant, given that it likely facilitates cultural identity and evolution (Tomasello, 2003; Henrich, 2016; Moffett, 2019). In Levin's framing, this expanded our cognitive light cone, albeit not yet in the technically transcendent angular shift necessary for objectivity, it does provide a furtive field doing so.

Evolutionarily, this is adjacent to primate vocalizations, e.g., the chimpanzee pant-hoot. Some researchers suggest this vocalization is used to generate in-group cohesion (Moffett, 2019). However, unlike human accents there is evidence that the individual differences in this chimp vocalizations are greater than group (Desai et al., 2022), aligning with Moffett's claim that chimp social units are based on individual intimacy. Hence the pant-hoots are not dialogic in chimps because their groups depend not on accents as a signal of in-group membership but direct indication of a familiar conspecific. This also aligns with cross-species vocalization research led by Jarvis, who finds that vocal production is only possible with predators. Hence our meek ancestors were able to adapt accents as dialogue largely because vocal variation was limited and hence was reliable as a signal of provenance (Jarvis, 2006), that further suggested adherence to normative expectations and social behavior (McElreath et al., 2003).

Transitioning to anonymous social units for a complex social organism that evolved from a species that were intimate with all social compatriots requires something like *synthetic intimacy*, i.e., they are like me or people with whom I an intimate, can identify with, and can trust to adhere to behavioral expectations McElreath et al. (2003), like food sharing, collaboration during hunting and gathering, cooperative breeding practices, etc. This creates a vulnerability, i.e., potential unwelcome social surprise that would need minimization in order to ensure the continued in-habitation and cooperative gain of an anonymous social unit (Section 5). Explicating emotional states and social norms using what linguistics call a “performative utterance” (Austin and Urmson, 2009; Searle, 2010b), provides both with a likely starting point for our cultural identity and symbolic communication that would have been important for our ancestors (Section 3.1).

Expressing *belief* in expectations as objective, i.e., presented *as if* from a neutral bystander, offers a minimal modification of vocalizations seen in our primate relatives yet sufficient to affirm an anonymous social unit. Our vocalizations, of course, played a large role both in the declaration itself where we began by labeling actions as *wrong* and as an initial signal of in-group or shared identity (of course, wrong just means surprising in this encultured, co-constructed, niche). The latter established an expectation of cooperator while the former allowed us to minimize the social surprise that followed acts seen as deviant. This constitutes an explication of belief in both the Bayesian statistical sense traditionally employed by the FEP (Section 2) but also tethers the statistical concept to our common understanding of faith in a culture, supernatural entity, or a universal moral code.

Harnessing the energetic process of a complex symbolic reality is sufficiently and parsimoniously achieved through a declarative, or explicit, belief in relatively egalitarian rules, i.e., policies that place a minimum value on all participants. Objective, symbolic, and “golden” (i.e., treat others as you would like to be treated) rules affirm the symbolic cultural identity that is likely the most ancient and foundational form of human morality as distinct from behavioral norms of other animals (Pfaff and Wilson, 2007; Boehm, 2012; Burkart et al., 2018). Logically, objectivity goes hand in hand with egalitarianism, given that a rule that applies to everyone implies that everyone (i.e., within-group) is, in at least a narrow sense, equal under the *law*. This view also aligns with Lorenz (1974) claim that hunter-gatherer groups generally have a word for in-group members of their tribe that corresponds to human and non-human. Thus, in this view, shared belief, egalitarian norms, and the very signified conception of humanity, i.e., *human beings*, represent both an agent's *moral circle* and at least an important foundation for our symbolic capacity.

This thesis has precedence in and implications for moral philosophy. A belief in objectivity reconciles Søren Kierkegaard's challenge to Hegel's argument that the power of objective rationality was sufficient to progress moral truth and practices further by insisting that objective analysis is (at most) backward looking while forward action requires faith (Halvorson, 2022). Belief need not be in a religious doctrine or a supernatural entity, shared faith in the pursuit of quest for moral rules and principles can be sufficient to guide moral progress. In other words, the very belief in objectivity can and does encourage us to find the necessary common ground to discover it. This also follows Nietzsche's critique of Schopenhauer's pessimism, i.e., his claim that morality is essentially negative. We inhabit symbolic selves that can come into being by abstracting negative behavioral norms, hence the underlying motivation is a positive construction and affirmation of identity. Also, given our morality ultimately follows from dyadic social connections, the emphasis of some moral philosophers, e.g., Socrates and Zhuangzi (Section 6), upon the relationship of love and moral behavior or virtue is bolstered.

## 2. FEP and Markov blankets

Before proceeding to unpack the FEP in an encultured setting, it might be useful to review its elements. The FEP offers a simple—perhaps almost tautological—description of self-organization. It starts with the notion of *things* that can be individuated from everything else in virtue of possessing a Markov blanket (Parr et al., 2020). This blanket provides a boundary that separates the interior of something from its external milieu (e.g., a particle within an atom, a fish in water, and a cat on dry land).

The very existence of a Markov blanket licenses an interpretation of the internal states as parameterizing probabilistic (i.e., Bayesian) beliefs about external states of affairs (Ramstead et al., 2023). This follows from the fact that, when conditioned on the Markov blanket, internal and external states are independent. This means for every external state there is an expected internal state and vice versa. In consequence, expected internal states can be read as encoding Bayesian beliefs about external states. Wherein external states influence internal states and are subsequently influenced through intermediate sensory and action states. For example, homeostasis ensures that a body's temperature is in predictable ranges relative to its environment.

The dénouement of this physics of sentience or Bayesian mechanics is that anything that exists can be read as if it is trying to infer the causes of sensory impressions on its Markov blanket (Ramstead et al., 2023). This can be viewed as a tendency to minimize surprise (i.e., variational free energy) or, equivalently, maximize (Bayesian model) evidence for an implicit world or generative model entailed by the internal states. This is sometimes known as self-evidencing (Hohwy, 2016). In short, every thing or object can be understood as acting and perceiving to minimize surprise or maximize predictability.

It follows that all choices and decisions are in the service of minimizing expected surprise (i.e., expected free energy), which has an interesting mathematical interpretation: expected surprise—or uncertainty—is entropy in information theory, which means that we are compelled to resist the second law of thermodynamics in virtue of minimizing uncertainty. This can be formalized by decomposing expected free energy into two parts (Da Costa et al., 2020). The first corresponds to expected information gain, which is the objective function for Bayes optimal experimental design (Lindley, 1956; MacKay, 1992); namely, the intrinsic or epistemic value that underwrites exploration and information-seeking behavior (Schmidhuber, 2006; Friston et al., 2017). The second part can be read as expected value, where value is just the log probability of preferred or characteristic states. This part corresponds to the extrinsic or pragmatic value that underwrites preference-seeking behavior (Attias, 2003; Botvinick and Toussaint, 2012; Lanillos et al., 2021). So, how does this help in terms of communication and culture?

Consider an ensemble of particles or a community of conspecifics. Each member of the ensemble is trying to minimize their free energy and—in so doing—minimize the collective free energy of the community in question. Technically, this takes us into the world of Markov blankets in the sense that if a community is a thing, then it has a Markov blanket (Palacios et al., 2020). Indeed, the very existence of Markov blankets at nested scales speaks to the scale invariant application of the FEP—an application that mandates the collective resolution of uncertainty. This has been used to useful effect to understand cultural niche construction in the context of the hierarchically mechanistic mind and active inference (Ramstead et al., 2018; Badcock et al., 2019; Vasil et al., 2020).

Many aspects of the active inference entail exchanges across our Markov blanket in terms of questions and answers—i.e., communication—in the service of resolving uncertainty (see Friston et al. (2015, 2020) for numerical illustrations). This speaks to theory of mind and the cohesion of Markov blankets that follows from rendering everything mutually predictable. Technically, this is sometimes read as a form of generalized synchrony (Friston et al., 2015) or distributed cognition. Note the emphasis here is on working toward a shared narrative or common ground (Tomasello, 2016), allowing me to predict you—and therefore minimize surprise. In other words, it paints a cooperative picture of collective self-organization in relation to adversarial and exploitative constructs seen, for example, in game theoretic accounts which assume that self-interested individuals only cooperate when there is clear evidence of extrinsic benefit (e.g., fitness, caloric, monetary). These accounts often fail to understand that intrinsic motivation of agents is due to the implicit assumption of fixed and static boundaries around the agents, rather than accepting the mutable and multiplicative interpretation of identity or “selfhood” (Section 3.2), one that extends to moral communities (Curry, 2016).

However, this is not the full story, it is clearly the case that people, institutions and cultures do not necessarily converge on the same world model. This is evident in all scales and levels of life and points to the complexity inherent in occupying numerous and hierarchically nested blankets, requiring coordination and prioritization of lower-level needs that gives rise to emotions and subjective conscious experience (Solms, 2019). Moreover, this may also reflect the fact that the world is constantly changing, therefore, providing the epistemic affordance that renders us curious creatures.

In a cultural setting, this means that there will always be an imperative to resolve uncertainty about others, in the service of being able to explain or predict their behavior (e.g., through stereotyping): an important aspect of active inference is to find the simplest account for another's behavior, in terms of their kind or type (e.g., does this person “speak my language” or “sound like a familiar person”). There is a deep mathematical reason for this tendency to explain self and others in simple coarse-grained terms, which follows from a decomposition of free energy into accuracy and complexity (Da Costa et al., 2020). In short, in building our encultured world or generative models, we will try to provide an accurate account of the sensed world that is minimally complex in accord with Occam's principle and try to share that worldview with everyone we choose to engage with (Albarracin et al., 2022). In what follows, we will unpack the ensuing imperatives for sentient behavior in terms of cultural transactions to explore the proposal that humanity's symbolic capacity is underwritten by belief in an abstract morality, i.e., in a myth of objectivity.

## 3. WEIRD null hypotheses

The mission the FEP sets forth is to pinpoint the fuzzy, mutable, and multiplicative boundaries and common shared frames of reference (or subjective context) that make up the structures of our world (Clark, 2017).^4^ The FEP allows us to reframe emotions, norms, societies, and language, that were initially approached with assumptions of rigid individuation that emphasizes extrinsic motivation to the inclusion of the psychologically innate (Noble, 2017; Kay and King, 2020), as a statistical imperative to maintain coherence while cohabiting numerous nested Markov blankets at various scales.

The reductive tendency however is important for this analysis primarily for two reasons. Firstly, one tried and true method for fulfilling the FEP's mission is by *deconstructing* the boundaries erected by more reductionist-oriented ideologies. Many researchers have effectively used static systems as *null hypotheses* to offer contrasting theories that imply sounder ontologies. Secondly, the analytical tendency is not unique to WEIRD societies but also shared by our nomadic ancestors, a tendency necessary for a species capable of reducing the natural world into discretized sounds (i.e., words). Words expand the normative framework that follows harmonious expectations of an expanded social unit or enjoined blankets by creating modular, mutable, and multiplicative symbolic structures.

### 3.1. Deconstructing symbols

The philosopher Jacques Derrida's terms “trace” and “transcendental signified” capture how humanity actively knits Markov blankets around shared beliefs in objective morality. In an effort to define the foundational structure of language he (unwittingly) aligns to the FEP, noting how words gain meaning from distinction but are essentially modeled off the linguistic, cultural, and natural environment from which they emerge. Much of the context of Derrida's work was a critique of the structuralist school's alleged treatment of language as a static, atemporal, and a historical structure that existed outside of speakers themselves. Particularly the approach of structuralist Claude Lévi-Strauss to language is analogous to single equilibrium analysis in physics, i.e., seeking to resolve uncertainty about what words could mean (Derrida and Bass, 1998).

Instead, Derrida returns to and expands on de Saussure et al. (1986) assertion that words gain their meaning from a play of differences, wherein the inherently fuzzy boundaries of signified conceptions are most visible when juxtaposed to their opposites, i.e., the word *cat* is somewhat meaningful as it is different from *mat* but more meaningful when contrasted with *dog*. However, this latter example only offers clarity for interpreters who are sufficiently acquainted with cultures wherein the top two domestic pets are cats and dogs and contrasted frequently in media portrayals and common idioms (e.g., “fighting like cats and dogs”). Moreover, the structure of each word (or morpheme) itself is possible because it conforms to the conventional norms of (some local version of) English and if spoken would be pronounced in an accent indicative of a dialect, etc.

Meaning is continually modified by the more immediate context that precedes and succeeds it in a given sentence, speech, or text. Thus each morpheme is itself a “trace” of the various threads of the individual, linguistic, and more broadly cultural contexts from which it emerges (Derrida and Bass, 1998, 2017; Collins and Mayblin, 2011; Derrida and Spivak, 2016). In FEP terms then, each signified conception is not a fixed structure demarcated with a stationary partition; words are fuzzy models of a particular symbolic environment constructed within the mind of a particular interpreter, where distinct textual or audible signifiers, along with the conceptual “binary opposition,” e.g., cat-dog distinctions of the signifieds are analogous to free energy minimization at nonequilibrium steady-state in physics.

Framing linguistic structures according to FEP terms and adopting a technically transcendent view of Levin's light cone as the dynamic scope of Self (see Section 1), suggests that the conceptual chain ultimately gains coherence as a hierarchy of conceptual identities, each with their own self-evidencing agenda, including a level that represents the cultural niche itself. As a psychoanalyst, Jacques Lacan studied the pathologies that befall those who reach adulthood with poorly developed self-concept, i.e., those who fail to inhabit a part of their respective cultural identity. Moreover, any participation in a symbolic or social reality demands its participants maintain some level of coherence in the web of symbols bound by innumerabledeontic conventions conventions. How do we ground an identity that is essentially symbolic?

Derrida's solution lies in his allegation that not only structuralists but much of Western (WEIRD) metaphysics amounts to an attempt to halt the otherwise endless conceptual pairing and linking chain using one central signifying concept, the “transcendental signified,” i.e., a single concept that holds the center of gravity and acts as the ultimate source of meaning and inviolable truth. This reductive, or what Derrida called the “logocentric” bias treated, in effect, an inherently steady state system with fuzzy and mutable boundaries as stationary, allowing philosophical, legal, moral, and even mundane arguments to yield to the invocation of a central cultural myth such as *God* or *democracy*. Viewed in FEP terms, culture itself represents the superordinate identity and all concepts are subordinate to its ultimate authority.

However, the transcendence could also seemingly be more trivial and even neutral, that emerges within the context of a philosophical claim or general argument, i.e., implied axioms. Derrida treated this as a bias that follows the difficulty in making *objective* claims. Transcendental signification, however, may be more fundamental to the structure of language than Derrida emphasized. It is not just a WEIRD metaphysical bias; it is a bias necessary for our symbolic capacity. Symbolic communication represents channels that allow speakers the chance to exchange thoughts, ideas, etc. by relying on a shared superordinate identity, one that itself requires deontic affirmation. According to the view of “cooperative communication,” the symbolic world, i.e., the social reality we each create with language, is one enjoined to others, founded on our psychological motivation to align mental states (Tomasello, 2016; Vasil et al., 2020). This can include sharing attention over common objects and events and responding to them similarly, at times with anonymous (though likely compatriot) conspecifics. This follows from the “adaptive prior” beliefs that we inhabit similar niches to certain conspecifics that may be otherwise anonymous (Vasil et al., 2020). Asking for directions, the time, or weather updates aligns the priors of our generative models with our often anonymous interlocutor.

Most viscerally, however, sharing an emotional response while “gossiping” about a neighbor's extramarital affair or a local news story of political corruption, while seemingly trivial exposes the motivation to affirm the integrity of the social reality the speakers cohabitate by calling attention to someone who violated a foundational norm (Dunbar, 2004; Hartung et al., 2019). This establishes a communicative “common ground” that implicitly assumes a shared cultural identity and promotes the explicit semantic analogizing of a common symbolic Self (Tomasello, 2016).

This would have been helpful for our ancestors who occupied a dynamic nomadic social niche and consequently shared the WEIRD bias for analytical thinking (Henrich, 2016), i.e., the myth of objectivity and the practice of discretizing reality by employing fuzzy boundaries around sounds was motivated largely by the need for common constraints around behavior. Moreover, the ultimate driver of the human analytical worldview was the normative frameworks that arose in response to the ecological and social challenges common generally to nomadic hunter-gatherers that shifted only following the move to agriculture and a relaxation of individual normative expectations and a strengthening of clans and other collectives with a more rigid social hierarchy. A species needs to suffer from a reification fallacy (as “misplaced” as it may be, see Whitehead, 2010), in order to embark on the road of symbols and social reality. In order to take a technically transcendent view and shine Levin's cognitive light cone over our cultural niche (see above). In Henrich (2020) words (p. 224):

Mobile hunter-gatherers, who possess extensive (not intensive) kin-based institutions, are field-independent [an indicator of an analytical vs. gestalt bias]. Anthropologists have long argued that, compared to farmers and herders who have more intensive kin-based institutions, hunter-gatherers emphasize values that focus on independence, achievement, and self-reliance while deemphasizing obedience, conformity, and deference to authority.

It follows that all of humanity is constitutionally disposed to construct hierarchical taxonomies, i.e., we are all to varying degrees WEIRD scientists but perhaps with a paradoxically mythical grounding. We are motivated to label in-group conspecifics with moral concepts in order to update our beliefs about the beliefs of our compatriots. The simplest view of the Markov blanket is that it is a common constraint on movement, it aligns internal constituents, e.g., organelles within a cell's membrane, tissues, and organs within a body, etc., against the exterior environment. Providing the interior with evidence of a cohesive self (Hohwy, 2016). Generally, norms follow the constraints that are a consequence of a shared identity. Employing a speech act to explicitly declare an act a norm violation reverses this causal sequence, providing evidence of a shared belief in behavioral constraints that imply a natural allegiance to an expansive, cultural identity—one that underwrites the very use of language generally. We can only participate in what Ludwig Wittengestein called “language games” if enough people with sufficient fidelity mythologize the rules of the game (Wittgenstein et al., 1969).^5^

### 3.2. Selfishly expanding the self

The critique that structuralism seeks to impose static, atemporal structures on language is a common refrain that alleges WEIRD societies are biased toward a reductive state-oriented metaphysics that seeks to resolve the uncertainty of biological agents, economic systems, or even the psychology behind human behavior. This opposes the more expansive view the FEP warrants which is that natural and cultural evolution progress by continually enjoining the blanketed boundaries of selves. This is what he called logocentrism, or what is most, in line with the FEP focus on beliefs, the myth of objectivity.

The neoclassical economic model of selfish, utility maximizing agents with stable preferences attempts to resolve uncertainty by assuming a singular equilibrium system that efficiently distributes scarce resources using market prices that signal and satisfy those preferences (Friedman, 2008; Jehle and Reny, 2011). However, economists since Keynes (2008) and Knight (2014) have argued that this reductive model leaves no room for evident economic phenomena including growth, recession, or even firms and their branding (Kay and King, 2020).

What Homo Economicus has afforded economists, however, are “neoclassical market failures” (Cowen and Crampton, 2003), that perhaps best demonstrates the ultimate utility of overly reductive analysis as an analytically potent null hypothesis demarks the necessary features of our social reality. Externalities, which define costs and benefits that are not internalized to a given agent, have been invoked, for instance, to explain persistent pollution and even economic growth, e.g., investments in *research and development*, education, and job training can only be partially captured by the initial proprietor, allowing the benefits to spill over into the wider society (Romer, 1994). Intriguingly, similarly to Derridean transcendental signification, economic structures are ultimately founded on the belief in shared adherence to deontic or ethical norms, i.e., “social trust,” that not only permit self-seeking behavior using market prices but ultimately allow individuals to transcend individual perspectives and interest and inhabit social levels of firms, nations, and communities (Fukuyama, 1996; Carney, 2021).

The point here however is not whether these indictments accurately capture the beliefs of any or the most notable adherents of these paradigms in these polemics, but the admittedly extreme reductionist caricature of these paradigms are precisely what allowed their detractors to emphasize the fuzzy boundaries, attractors, and overall dynamics that exist in a complex world of steady-state equilibria and ultimately irreducible uncertainty. Oliver Scott Curry likewise uses Game Theory to define morality across human cultures as a cooperative force within symbolic groups such as culture and kin (Curry, 2016). This ability to expand the interiors of the self can also be described as overcoming another neoclassical market failure, “information asymmetry” (see Hidalgo, 2015). Arrow and Akleroff demonstrated the problems of uncertainty between producers and consumers in healthcare and used car markets respectively. Healthcare costs can be difficult to manage and buying used cars is challenging because the agent or entity that possesses or produces market goods always has more information than potential buyers (Akerlof, 1970; Arrow, 2004).

These problems in principle exist in every market, hucksters, snake oil salesmen, and other potential deviants always and everywhere threatening to exploit economic commons to personally benefit but lower the overall trust in the marketplace as a whole. These problems are overcome with company branding, government regulation/accreditation, uniforms, etc. Importantly, these represent a promise of future behavior, or contribute to the “regimes of expectations” within the FEP framework (Constant et al., 2019).

The competitive, cooperative, and collaborative dynamics correspond to the distance of the numerous social identities we occupy that serve to overcome information asymmetries and the levels of “social trust” we place within them (Fukuyama, 1996). Information asymmetry is also present in dyadic or intimate relations in the case of the “principal-agent” problem, how to trust someone to manage someone's business or assets to act in the best interest of the proprietor. This highlights the inescapable deontology or, at its core, simple morality that ultimately grounds market transactions of any scale and inclusive of intimate or anonymous participants.

Perhaps the most telling market failure, as Ronald Coase pointed out, if prices can efficiently signal value and motivate the movement of scarce resources there should be no need for firms, particularly the large, bureaucratic entities we commonly observe in the marketplace (Coase, 1937). Simon (1992) offered a plausible solution to the ultimate failure of the neoclassical market paradigm, i.e., to align the otherwise disparate goals if autonomous agents (p. 78):

[Company Employees] perceive the organization's goals as their goals—which is precisely what identification means. “We” and “they” are fundamentally important pronouns in the language, and an individual's conception […] of who “we” are defines his or her frame of reference in making decisions.

Just as our civic institutions, including churches and schools, generate general social patterns of behavior by structuring with and around individual self interests, economic institutions *transcend* what neoclassical school has traditionally taken as the lodestone of the economic agent, private self interest. The firm exploits our ability to inhabit social selves that requires mutual social trust both externally and internally. Employees need to have trust in co-workers and the firm's hierarchical structure in order not just to receive a paycheck but to freely contribute time and energy and align what may be otherwise disparate preferences and goals with those of the firm, and portion of the firm that they are employed. Other companies and customers need to trust firms and their branding to engage and purchase their products. This demonstrates that firms exist largely within the social commons. An idea supported by the notion of *corporate identity* (Balmer and Greyser, 2003).

Though modern companies exist in a web of social institutions that include constitutions, representative governments, legal precedence, etc. that are products of complex historical efforts to establish common social identities, it still may be tempting to see them as vehicles for unvarnished self-interest. However, social group identity theory details how people, particularly in times of uncertainty, are prone to joining malevolent groups such as terrorist organizations and criminal gangs despite the violation of self-interested motivation (Hogg, 2000). Revealing that without the presence of *prosocial* group labels humans tend toward shibboleth in its more raw, biased form.

Modern educational, economic, and political institutions are far from the only extant consequence of humanity's need to find meaning and self identity as well as minimize uncertainty within groups and the connection with members therein. The most psychologically potent exploitation of groups remains religion. According to Alcorta and Sosis (2005), religion has much in common with social rituals in non-human animals but distinct in cultivating shared emotional responses to abstract symbols. Religion, in their view, serves both as an *extrinsic* signal of one's identity for others as well as an *intrinsic* motivation to find one's own identity, particularly during adolescence. Through rituals, particularly during adolescence, nomadic hunter-gatherers facilitated the alignment of members into a broader cultural identity in order to face a range of broad ecological problems and norms that would ideally be fit to serve the group in such circumstances (e.g., individual vs. collective, see Greene, 2013). Religious and moral communities, companies, countries, and cultures all converge on the common tendency to produce transcendental signification or mythologize belief in objective norms in order to foster an individual's self-belief as part of a greater whole.

### 3.3. The feeling of objectivity

The fuzzy nature of FEPs treatment of the Markov Blanket and scale invariance of uncertainty minimization allow us to highlight the imaginary boundary that partitions humanity from other animals and identify the contiguous slope from which our ancestral trajectory began. Although this symbolic dimension of our psychology is uniquely practiced by humanity, the mechanisms that innately, impulsively, and instinctively drive us to do so are likely more common in the animal world. Mark Solms employed the FEP to argue the presence of emotions themselves gives rise to consciousness. Monkeys, dolphins, elephants, and even fruit flies operate in sufficiently complex behavioral patterns that require active and rapid prioritization, emotions afford organisms precisely the management function so demanded. In his words, (Solms, 2022, p. 194):

[Emotions offer a] capacity to compartmentalise, [enabling] the system to rank its needs and their attendant predictions (i.e., the salient sources of expected free energy) categorically, over time, and to focus its computational efforts upon the prioritised compartment. This is the statistical-mechanical basis for the observed fact that each affect possesses not only a continuous hedonic valence […] but also a categorical quality […] they possess both quantity and quality. […] affects are always subjective, valenced and qualitative. They have to be, given the control problem they evolved to handle.

Researchers Anderson (2022) and Adolphs (2013) formalize the notion of emotions as an “intervening variable,” i.e., mental states that can be triggered by a variety of stimuli and ultimately evoke a variety of different responses. A shadow from above can either elicit no physiological response, or trigger fear in a mouse. The mouse can respond by fighting, freezing, fleeing, etc. Intriguingly they partly define behavior as indicative of emotional brain states by contrasting them with another reductive, objective oriented framework, behaviorism, that reduces behavior to stimulus and response (Anderson, 2022). In Anderson and Adolfo's framework, behavior that meets a number of dimensions that indicate deviations from a purely physiological response, i.e., reflexes or conditioned responses which were thought sufficient to account for organic behavior in Watson and B.F. Skinner's view, suggests the presence of an intervening brain state.

The view of intervening emotional brain states as management tools of competing priorities aligns with humanity's moral practices. Morality, in this view, is simply a symbolically scaled version of the hedonic valence (pleasure vs. unpleasure) animals feel in order to persist, i.e., how they continue to carry out their relative pursuit of evidence to verify the belief in their own existence. Our impulse to sacrifice ourselves in war to protect our country or tackle a thief or simply watch morality-laden media, e.g., Law and Order SVU (see Knop-Huelss et al., 2020), speaks to either an implicit or explicit belief in a broader, and symbolic identity and the adherence to objective norms on which it is reified. A make believe self that, given the decomposition between extrinsic and instinct motivation to minimize uncertainty (Friston et al., 2015), can either be seen as authentically altruistic or cynically signaling, or at any point in between. Behaving in compliance with moral norms or simply uttering support of them (e.g., gossip, see Dunbar, 2004) provides evidence of a symbolic identity that offers structure and stability for constituents and generally fosters a constructive feedback loop.

It is precisely the objective, i.e., neutral, third-party perspective that both distinguishes humanity's moral practice and enables our extensive and anonymous social existence (Burkart et al. 2018, p. 9). According to Burkart et al. (2007) many of the foundational traits of our moral practice are found in other species including our primate relatives, particularly those that engage in cooperative breeding such as marmoset and callitrichid monkeys display “unsolicited prosociality,” i.e., they spontaneously cooperate without the assurance of reciprocation (Burkart et al., 2007). Chimps, our nearest relations with whom we share a common ancestor, do not rank high in terms of other-regarding behavior but even they display implicit rules such as a prohibition against infanticide and even experience arousal when the victim is an in-group member. They pay cognitive attention (stare) to those who violate the “putative” norm regardless of group membership and experience heightened arousal when the victim is an in-group conspecific (Burkart et al. 2018, p. 6).

Bekoff and Pierce (2009) cite the observations of evident compassion and sympathy animals display in the wild and captivity to argue that morality is not an exclusive human enterprise. However, they explicitly note that if our definition of morality includes the use of symbolic rules then it becomes exclusively human. The trouble with this carve-out, however, is that according to Schein and Gray (2018) human moral practice, while it includes common elements we share with other animals such as beliefs, emotions, and sociality, for humans it also demands a symbolic dimension. It is the abstraction of the implicit rules, the drive to take a neutral stance (even if we retain a parochial bias for intimates and kin), that makes morality fundamental for our social and symbolic existence.

Moral objectivity additionally goes hand in hand with a “golden rule” or egalitarian moral emphasis. According to researchers Chris Boehm and David Pfaff, the most foundational, ancient, and preferred human moral system supports a flat hierarchical structure. Simply put, a mental model where everyone is treated equally is an easier sell for existing and potential inhabitants. In FEP terms, sharing a generative model that places a minimum value on all participants, as opposed to one that more aggressively prioritizes some agents over others, presents an alluring model to encourage adoption and alignment of the model, and hence a mutually supportive feedback loop.

## 4. Beliefs by babel

*Change your tone and shape so often that they can never categorize you*

- Charles Bukowski

Our adaptation for prior beliefs that align our cognitive perspective with anonymous conspecifics (see Vasil et al., 2020), was likely cultivated in the evolutionary expansion of “in-group favoritism” (Masuda and Fu, 2015), using a tag based social unit (Cohen, 2012; Moffett, 2019). Specifically one that responds to what Derrida called our phonocentric bias, i.e., our voice, as evident in our accent bias that emerges early in infancy (Kinzler, 2021). Within the context of depersonalized, stereotyped groups along with our own self-conceptualized roles and identities therein (Hogg, 2000; Burke and Stets, 2009), the need to call attention to a norm violator and share in a state of moral outrage would not only be an extremely high-priority speech act, but it would also serve as what Searle (2010b) calls a “constitutive” act, a symbolic communicative event that brings about a structure that would not otherwise exist.

Chimps and other animals with comparable cognitive capacity cohabit with intimates. Individuals can exist in a stable environment among the vicissitudes of environmental uncertainty. As their social units grow to include more members than an individual can keep tabs over, the group tends to fracture (Moffett, 2013, 2019). Demonstrated above is humanity's ability to effectively synthesize this intimacy, we construct generative models of the behavior of innumerable conspecifics beyond those we are familiar with based largely on branding, insignia, uniforms, and finally accents. A process that involves, at times explicitly (e.g., fictive kin) paradigmatic analogy (Hofstadter and Sander, 2013).

The most innate and impulsive method of interpersonal synthesis is documented in research on social bias, e.g., stereotyping, entitizing, depersonalizing, etc. A topic that defies our modern rational expectations and a practice we hope, often for important historical reasons, we could extinguish. However, as Kinzler noted this bias is a, or maybe the, double edged sword of humanity. It is at the foundation of our desire to make meaningful connections (Kinzler, 2021) and thus points to the developmental and evolutionary origins of our symbolic semiotic capacity. While our ability to extensively cohabit with anonymous individuals may be a consequence and not a cause of our symbolic capacity, it seems more likely that the former acted as a bridge to the latter. Infants display early signs of expansive prosociality and moral preferences (Warneken and Tomasello, 2009; Bloom, 2013), and they also display signs of group bias (Kinzler, 2021).

This is further suggested by the limits of human innate moral or altruistic impulse. Researcher Bloom (2013) notes that while infants display “certain moral foundations” (p. 8), including prosocial behavior, their kindness is bounded. Bloom argues for *contingent altruism*, the idea that fitness maximizing behavior can be cooperative rather than harmful or competitive but is biased. This bias, as we saw with in-group bias in primates generally, can be genetic/inclusive as in ants or bees, can be based on familiarity or intimacy as in primates generally, or can be symbolic as in human groups. One interim step that sets the social milieu of this hypothesis of our origins of symbolic groups is suggested by our vocals. Cognitive Anthropologist Cohen suggests that Game theory simulations show how tag-based simulation can achieve contingent altruism or expand our group size. In her words (Cohen, 2012, p. 588):

Existing evidence warrants the preliminary conclusion that accent markers meet the demands of an evolutionarily viable tag and potentially afforded a cost-effective solution to the challenges of maintaining viable cooperative relationships in diffuse, regional social networks.

Our ancestors along with extant hunter-gatherers occupied social units that expanded well beyond intimates and kin. Hill et al. (2011) analyze extant hunter-gatherer communities and find that most individuals in residential groups are genetically unrelated. Mark Moffett goes further and asserts that included within the fuzzy boundaries of a typical hunter-gatherer society are thousands of individuals that extends well beyond not only kin but intimates. This suggests anonymous societies are a distinct and universal feature of human social existence. As Moffett (2019) describes (p. 116):

Numerous accounts tell how hunter-gatherers of recent centuries felt secure in the presence of their “own kind.” Asked who they were, typically a hunter-gatherer would give you the name for a community encompassing several, often a dozen or more, bands spread over a wide geographic range. These band societies had populations ranging from a few dozen to perhaps a couple of thousand individuals.

Anthropologists have long focused on identity signals as a possible origin of our symbolic behavior, one prior to speech, art, and language. The ubiquity of body ornamentation and hair dress, for instance, across human cultures including extant hunter-gatherers along with its ancient discovery, and finally the evolutionary utility identity signaled would have served makes it an attractive candidate for the origins of human semiotics. Particularly the use of red ochre dye and its frequent use to signal cultural identity (Henshilwood et al., 2011).

Given evidence from modern humanity along with pan, our nearest extant primate relatives, the tag we first employed to facilitate this transition was likely our voice (Cohen, 2012; Moffett, 2013, 2019; Kinzler, 2021). Although accents are now by-products of our symbolic system of speech, they are adjacent to any general vocally produced Shibboleth that, at its core, is not symbolic. Nor did accents necessarily evolve as an identity signal, conditions that fostered capacities for vocal imitation and production such as social learning and communication later could have afforded our ancestors the opportunity to exploit it later as one. Cohen suggests the dynamic of population isolation, dispersal, and drift would have raised the likelihood of accents as an identity maker.

She argues that accents possess a variety of features that make them a promising candidate to overcome many of the common objections to tag based cooperation while offering as a bridge, i.e., a cognitive “mill” in Heyes (2018) preferred terms. These include salience (easy to notice), honesty (hard to fake), discernible, and dynamic (allows for numerous accents based social categories, e.g., idiolect, sociolect, etc.). For instance, their salience is evident given that children as young as 6 can discern a speaker's provenance before they complete a single word. Accents furthermore map to social and economic class and demarcate social identity more generally (Moffett, 2019; Kinzler, 2021).

From an FEP perspective this allows individuals to use accents to actively and parsimoniously infer one's identity, promoting the likelihood that the phenotype and cultural incentive for vocal learning remains high. Moreover, the continuous nature of accents, wherein someone growing up in Rhode Island will sound distinct from someone from Boston, but both will sound more alike than someone from Canada. Accents remain a reliable signal of provenance given the difficulty it is to acquire a new one past childhood. This example also covers the *discriminable* quality as well.

Regarding the ancient criteria, the population drift and dispersal that our ancestors endured during the adverse climate events of the African Middle Stone Age likely contributed to the value of accents at this juncture. Furthermore, the body ornamentation referenced earlier suggests identity tags stretch far into our evolutionary past (Henshilwood et al., 2011). Finally, with regard to ontogeny, as mentioned above Kinzler (2021) have noted that infants prefer their mother's vocals and those who speak in their mother tongue over foreign dialect and speech.

In addition, Jarvis (2006) notes that in species ranging from primates to birds, due to concerns related to exposure only predators can exhibit a high vocal production range, the type necessary for speech. As our ancestors were meek primates amongst fierce competitors and predators, their accents were likely a reliable signal of provenance and hence indicated adherence to certain social norms that would aid in minimizing surprise. As population viscosity increased due to environmental predation, vocals would reliably signal provenance and hence behavioral norms, hence the claim that modern ethnic centrism continues to functionally signal an overlap of norms and conventions (Boyd and Richerson, 1988). Moffett (2019, p. 29) uses the example of a prominent vocalization used in Apes to suggest a mechanism our ancestors may have employed to signal in-group vs. out-group identity, in his words:

[A] simple shift in how the apes use one of their vocalizations, the pant-hoot, could make that sound essential for identifying each other as society members. […] The cry expresses excitement among apes on an outing together, a yay team cheer that strengthens the bonds between males and helps parties within earshot keep tabs on each other. […] It's the pant- hoots from a foreign community that really agitate the apes.

## 5. Declaring deviance

Humans are caught […] in a net of good and evil. I think this is the only story we have and that it occurs on all levels of feeling and intelligence. Virtue and vice were warp and woof of our first consciousness

- John Steinbeck, East of Eden

The anonymous social units our ancestors inhabited through shibboleth were nonetheless vulnerable to the problem of fee-riders and deviants. The unexpected observation of socially deviant behavior of a compatriotic and anonymous conspecific required a general, rather than a particular, categorization. Our ancestors, instead of simply treating a deviant as an out-group member and thereby threatening the expectations of social harmony, applied a generalized label to make explicit the conflicting priorities that arise from inhabiting multiple blankets or social identities (individual, familial, cultural). As emotions manage our physical and social identities, thought and language may have emerged, following this account, to facilitate our adoption of novel symbolic ones.

Declaring that a conspecific occupies the role of a deviant and the subsequent surprise minimization, create an implicit belief in a shared symbolic identity. In linguistics, the evil tag represents a type of speech “declaration” known as a “performative utterance,” i.e., one that modifies the reality in which it was uttered (Searle, 2010b). “*You are fired*,” “*the meeting is adjourned*,” “*we are at war with France*” are all utterances that potentially modify the social reality from which it emerged. Moreover, evil represents an elemental form of a directive known as a “feature placement” (Strawson, 2011), this is a singular word directive that need not be symbolic and attached to uniquely human contexts, they are seen elsewhere in the animal kingdom. For example, the waa-bark and pant-hoot or vervet Monkeys above or below calls that signal specific types of predators influence their conspecifics and effectively social world (Wilson, 2020).

Hence this faculty was available to our pre-linguistic ancestors and may have been repurposed for psychological social construction. Declaring someone a villain gives them a new identity and implies that we exist in a shared community in which he played this novel role. After employing a modified version of a pant-hoot to signal identity, our ancestors employed a modified hominin version of a waa-bark (Boehm, 2012) to label anonymous yet ostensibly faithful compatriots as deviants. Non-believers that threatened the continuity of our fuzzy identities due to the negatively valenced affect the surprise aroused, accelerating our capacity for socially constructed affect (Barrett, 2017), i.e., pairing this vocalization with the unwelcome feeling of surprise. Thereby employing the “supra-communicative dimension” of language and bolstering individual abstract conception of the alleged deviant (Clark 2016, p. 282).

Although socially constructed affect likely emerged from causes that explicitly threatened individual survival, e.g., distress in the absence of a mother, given that social construction typically serves a wider social purpose (e.g., the typical example given of the phenomena is money), it is feasible that once established, higher order emotional capacity was exploited at the level of cultural identity. This would serve to discourage harmful in-group behavior, promote outward signals of allegiance, and cultivate the emotional and cognitive awareness of cultural identity.

Evil, wrong, and similar labels of moral concepts and types may have been fostered in the malleability of child's play. Mark Solms points to this emotion (Section 3.3) as the likeliest to be involved in the origins of human cognition. Play cultivates the mind at its most creative and inventive. While non-symbolic roles are creative, they are based on natural or observable models, i.e., playing as alpha or dominant vs. submissive. Playing as a deviant or as police/norm enforcer, while based on likely observations within the context of an anonymous social unit, necessarily involves essentially something unnatural: the will or agency to determine one's identity through action.

## 6. Conclusion: equivocal transcendence

*I know virtually nothing, except a certain small subject—love, although on this subject, I'm thought to be amazing, better than anyone else, past or present*.

- Socrates

*The Way is lost in the glorification of right and wrong. The Way is lost in the completion of love*.

- Zhuangzi

While there is likely an inherent limit to human knowledge that elides our pursuit of objective understanding, the FEP offers a forward epistemic trajectory through consilience, i.e., finding common patterns across various disciplines even if each is independently *incomplete*, as Gödel demonstrated. This application to morality, social science, and anthropology through symbolic semiotics demonstrates humanity possesses an existential freedom as licensed from the symbolic personal and social identity we inhabit, but also that along with this freedom is the imperative to fill that freedom, with faith. At minimum we require a belief in a technically transcendent perspective, that an objective viewpoint hovers over our social reality. This combines the privileged objective “spectator” view of the past, as proposed by Hegel, with Kierkegaard's “leap of faith,” which is necessary for forward-looking action (Halvorson, 2022). The most parsimonious statement of this faith is the belief that we are all parts of a greater whole.

Emanuel Kant and Arthur Schopenhauer both espoused a view of human morality as effectively negative, i.e., viewing them as constraints that impose a burden and thwart the instinctive desires of mankind to be free and in effect, happy. This implies that in the past there was an untrammeled human existence without such irksome impediments. Not only is this perspective unwarranted, but it can also be effectively reversed. Our norms, though negative on their own, are in fact more broadly creative aspects that, often implicitly and unconsciously, rely on an affirmative attachment, identification, or belief in a symbolic identity that, at minimum, exceeds the individual self. This feeling ranges from more quotidian and surface-level attachments such as aligning mental states using words as detailed above (Vasil et al., 2020), it also reaches more deeply, such as the phenomenon documented in neuroscience as a “transcendental experience” that is not only experienced through intimate social relationships but also when we identify with fictional or anonymous characters in stories (Fletcher, 2021). This emphasizes what Zhuangzi and Socrates alike understood, that to achieve morality we cannot stare exclusively at rights and wrongs; we need to be guided by underlying benevolent feelings that allow us to transcend our own identity and inhabit a broader, or even, non-self.

The simplest and perhaps oldest explanation of human morality, short of oversimplification (to follow Einstein's adage/Occam's principle), is that moral virtue represents behavior that serves a greater good while offering individuals the promise of purpose, meaning, status, and self-esteem that overcome the individual costs and risks associated with moral behavior. The symbolic self can be seen in several different frameworks: the Hindu notion of the individual “Atman” as bound within the structure of the body of a cosmic being “Purusha” (Eknath, 2007); Aristotle's conception of proper citizenship within a community; the union of the faithful with the God of Abraham and in the Confucianist and Taoist concept of Wu-Wei that defines morality as the spontaneous behavior that follows an authentic connection of the individual to the greater good (Ivanhoe and Van Norden, 2005; Slingerland, 2014). Virtue, in all these frameworks, is achieved when the individual identifies and serves a particular part of an abstract whole.

This confronts perhaps the final act in the litany of WEIRD Null Hypotheses, i.e., western moral philosophy. Both utilitarianism (the idea that computing the aggregate welfare of behavior is the ultimate determinant of moral value) along with deontology (the idea that morality boils down to adherence to preset moral principles) represent victims of the fallacy of misplaced concreteness in trying to absolve, a priori, the presence of uncertainty in the realm of moral decision making (Greene, 2013). While moral practices are concerned with aggregate welfare impact and employ principles to navigate the dictates of behavioral norms that do so—by attempting to compute specific rules and principles—these theories take too literally the myth of objectivity, essentially presuming the ability to overturn the Second Law of Thermodynamics, i.e., the non-diminishing character of uncertainty. Practically speaking, the problems morality addresses involve nuanced and complex ecological, cultural, and social contexts that can be known either ex-ante or ex-post.

Morality is best thought not as an objective function, wherein the rules themselves should be computed a priori, but as orthogonal dimensions of the subjective goals and desire. Although believing that each subject bears responsibility is central to both human morality and in turn, our distinctive symbolic capacity, it does so largely by reshaping our subjectivity. We become aligned to a nested web of groups, within which we impulsively cooperate, and beyond which we fiercely compete, but even then we are constrained by our higher order allegiances. Our passions for political parties is morally justified in so far as they are ultimately constrained by the desire to affirm our national allegiances; our corporate competitiveness is likewise so long as private interest is aligned with the longer-term interest of the market, consumers, and social stakeholders.

The experience of morality is not in the meticulous accounting of specific rights or wrongs nor in whether they conform to preset moral principles. Ultimately our symbolic morality and the human condition generally is an effort to overcome the informational asymmetries that are an inevitable and necessary consequence of the personal, social, and cultural boundaries that permit our distinctive existence yet the same boundaries through which we come to understand an important lesson of essences, concepts, or signification, which is simply that our uniqueness is truly convergent with the experience of universal connection. Both developmentally and historically we migrate from the technical transcend subjective perspectives that underwrite even basic symbol use to self-transcendence that allows us a *visceral* or physiologically felt connection to others and the wider world.

## Data availability statement

The original contributions presented in the study are included in the article/supplementary material, further inquiries can be directed to the corresponding author.

## Author contributions

SR: Writing—original draft.

## Footnotes

^1^Heyes describes the elemental mechanisms of human distinction as our cognitive “grist” rather than “mills.” The former are the causal factors whereas the latter are only subsequently made possible (Heyes, 2018). Cultural evolution, for instance, requires the ability to first acquire and share cultural information. Other grist candidates such as tool use, vocal communication, theory of mind (Heyes and Frith, 2014), and even social rituals (Alcorta and Sosis, 2005), appear distinct to humanity largely to the extent that they are symbolized, and hence are more likely mills.

^2^Under the FEP, Bayesian beliefs refer to conditional probability distributions encoded by the biophysical states of a particle or person. For example, neuronal activity might encode the average (a.k.a., expectation) of some probabilistic or fuzzy belief about the identity of a visual object causing visual sensations.

^3^Intimate relations here are contrasted with the conspecifics we know only through audio of visible markings or *insignia*. In humanity's case, we use accents, uniforms, and more recently online profiles. This type of social relation extends to non-human animals, for example, ants and bees (see Moffett, 2019).

^4^Markov blankets are equivalent to the “reference frames” that Einstein used in his formulation of general relativity. Before Einstein physicists distinguished between universal forces, such as gravity, and the fictional forces experienced by objects within an accelerating *frame of reference*. The theory of general relativity concluded however there is no privileged or observer-independent objective perspective (Einstein and Lawson, 1961). The notion of a reference frame extends other discipline's including Dennis Noble's Biological Relativity (Noble, 2017), to the modern understanding of linguistic communication in terms of “common ground” or shared prior semantic context (Seising, 2012; Tomasello, 2016), and to the lowest known levels of reality in mathematical formulations of quantum information theory in terms of aligned quantum reference frames (QRF) (Fields et al., 2022).

^5^Similar to Gödel's second incompleteness theorem, Wittengstein challenged the ability to mathematically pursue objective truth that Bertrand Russell, for instance, arguably did in Principia Mathematica in his “axiom of reducibility.”

## References

[B1] Adolphs R. (2013). The biology of fear. Curr. Biol. 23, R79R93. 10.1016/j.cub.2012.11.055 23347946 PMC3595162

[B2] Akerlof G. A. (1970). The market for “lemons”: quality uncertainty and the market mechanism. Q. J. Econ. 84, 488. 10.2307/1879431

[B3] Albarracin M. Demekas D. Ramstead M. J. D. Heins C. (2022). Epistemic communities under active inference. Entropy 24, 476. 10.3390/e24040476 35455140 PMC9027706

[B4] Alcorta C. S. Sosis R. (2005). Ritual, emotion, and sacred symbols: the evolution of religion as an adaptive complex. Hum. Nat. 16, 323–359. 10.1007/s12110-005-1014-3 26189836

[B5] Anderson D. J. (2022). The Nature of the Beast: How Emotions Guide Us, 1st ed. New York, NY: Basic Books.

[B6] Arrow K. J. (2004). Uncertainty and the welfare economics of medical care. 1963. Bull. World Health Organ. 82, 141–149. 15042238 PMC2585909

[B7] Attias H. (2003). Planning by probabilistic inference. Proc. Mach. Learn. Res. R4, 9–16.

[B8] Austin J. L. Urmson J. O. (2009). How to Do Things with Words: The William James Lectures Delivered at Harvard University in 1955, 2nd ed. Cambridge, MA: Harvard Univ. Press.

[B9] Badcock P. B. Friston K. J. Ramstead M. J. (2019). The hierarchically mechanistic mind: a free-energy formulation of the human psyche. Phys. Life Rev. 31, 104–121. 10.1016/j.plrev.2018.10.002 30704846 PMC6941235

[B10] Balmer J. M. T. Greyser S. A. (eds) (2003). Revealing the Corporation: Perspectives on Identity, Image, Reputation, Corporate Branding, and Corporate-Level Marketing: An Anthology. London: Routledge. 10.4324/9780203422786

[B11] Barrett L. F. (2017). How Emotions Are Made: The Secret Life of the Brain. Boston, MA: Houghton Mifflin Harcourt.

[B12] Bekoff M. Pierce J. (2009). Wild Justice: The Moral Lives of Animals. Chicago, IL: The University of Chicago Press. 10.7208/chicago/9780226041667.001.0001

[B13] Berger L. R. Hawks J. De Ruiter D. J. Churchill S. E. Schmid P. Delezene B.. (2015). Homo naledi, a new species of the genus Homo from the Dinaledi Chamber, South Africa. Elife 4, e09560. 10.7554/eLife.09560.031 26354291 PMC4559886

[B14] Bloom P. (2013). Just Babies: The Origins of Good and Evil, 1st ed. New York, NY: Crown Publishers.

[B15] Boehm C. (2012). Moral Origins: The Evolution of Virtue, Altruism, and Shame. New York, NY: Basic Books.

[B16] Booker C. (2004). The Seven Basic Plots: Why We Tell Stories. London: Continuum.

[B17] Botvinick M. Toussaint M. (2012). Planning as inference. Trends Cogn. Sci. 16, 485–488. 10.1016/j.tics.2012.08.006 22940577

[B18] Boyd R. Richerson P. J. (1988). Culture and the Evolutionary Process, paperback edition. Chicago, IL: University of Chicago Press.

[B19] Burkart J. M. Brügger R. K. van Schaik C. P. (2018). Evolutionary origins of morality: insights from non-human primates. Front. Sociol. 3, 17. 10.3389/fsoc.2018.00017

[B20] Burkart J. M. Fehr E. Efferson C. van Schaik C. P. (2007). Other-regarding preferences in a non-human primate: common marmosets provision food altruistically. Proc. Nat. Acad. Sci. 104, 19762–19766. 10.1073/pnas.0710310104 18077409 PMC2148372

[B21] Burke P. J. Stets J. E. (2009). Identity Theory. Oxford: Oxford University Press. 10.1093/acprof:oso/9780195388275.001.0001

[B22] Carney M. (2021). Value(s): Building a Better World for All. New York, NY: Public Affairs.

[B23] Carroll S. (2021). Michael Levin on Growth, Form, Information, and the SelfPodcast. Available online at: https://www.preposterousuniverse.com/podcast/2021/02/01/132-michael-levin-on-growth-form-information-and-the-self/

[B24] Clark A. (2016). Surfing Uncertainty: Prediction, Action, and the Embodied Mind. Oxford: Oxford University Press. 10.1093/acprof:oso/9780190217013.001.0001

[B25] Clark A. (2017). “How to knit your own Markov Blanket: resisting the second law with metamorphic minds: resisting the second law with metamorphic minds,” in Philosophy and Predictive Processing, eds T. Metzinger, and W. Wiese (Frankfurt am Main: MIND Group), p. 1.

[B26] Coase R. H. (1937). The nature of the firm. Economica 4, 386–405. 10.1111/j.1468-0335.1937.tb00002.x

[B27] Cohen E. (2012). The evolution of tag-based cooperation in humans: the case for accent. Curr. Anthropol. 53, 588–616. 10.1086/667654

[B28] Collins J. Mayblin B. (2011). Introducing Derrida: A Graphic Guide. London: Icon Books.

[B29] Constant A. Ramstead M. J. D. Veissière S. P. L. Friston K. (2019). Regimes of expectations: an active inference model of social conformity and human decision making. Front. Psychol. 10, 679. 10.3389/fpsyg.2019.00679 30988668 PMC6452780

[B30] Cowen T. Crampton E. (eds) (2003). Market Failure or Success: The New Debate, reprint edition. Cheltenham: Edward Elgar. 10.4337/9781781950005

[B31] Curry O. S. (2016). “Morality as cooperation: a problem-centred approach,” in The Evolution of Morality, eds T. K. Shackelford, and R. D. Hansen (Cham: Springer International Publishing), 27–51. 10.1007/978-3-319-19671-8_2

[B32] Da Costa L. Parr T. Sajid N. Veselic S. Neacsu V. Friston K. (2020). active inference on discrete state-spaces: a synthesis. J. Math. Psychol. 99, 102447. 10.1016/j.jmp.2020.102447 33343039 PMC7732703

[B33] de Saussure F. Bally C. Sechehaye A. Riedlinger A. (1986). Course in General Linguistics. LaSalle, IL: Open Court.

[B34] Deacon T. W. (1998). The Symbolic Species: The Co-Evolution of Language and the Brain, 1. publ. as a norton paperback edition. New York, NY: Norton.

[B35] Derrida J. Bass A. (1998). Positions, paperback ed. [nachdr.]. Chicago, IL: Univ. Chicago Press.

[B36] Derrida J. Bass A. (2017). Writing and Difference. London: Routledge.

[B37] Derrida J. Spivak G. C. (2016). Of Grammatology, fortieth-anniversary edition. Baltimore, MD: Johns Hopkins University Press. 10.56021/9781421419954

[B38] Desai N. P. Fedurek P. Slocombe K. E. Wilson M. L. (2022). Chimpanzee pant-hoots encode individual information more reliably than group differences. Am. J. Primatol. 84, e23430. 10.1002/ajp.23430 36093564 PMC9786991

[B39] Dunbar R. I. M. (2004). Gossip in evolutionary perspective. Rev. Gen. Psychol. 8, 100–110. 10.1037/1089-2680.8.2.100

[B40] Dupré J. O'Malley M. A. (2009). Varieties of living things: life at the intersection of lineage and metabolism. Philos. Theory Biol. 1, 1–3. 10.3998/ptb.6959004.0001.003

[B41] Earman J. The Society of Christian Philosophers (1993). Bayes, hume, and miracles. Faith Philos. 10, 293–310. 10.5840/faithphil19931039

[B42] Einstein A. Lawson R. W. (1961). Relativity: The Special and the General Theory. New York, NY: Crown Trade Paperbacks.

[B43] Eknath E. (ed.). (2007). The Bhagavad Gita. The Classics of Indian Spirituality, 2nd ed. Tomales, CA: Nilgiri Press.

[B44] Fields C. Friston K. Glazebrook J. F. Levin M. (2022). A free energy principle for generic quantum systems. Prog. Biophys. Mol. Biol. 173, 36–59. 10.1016/j.pbiomolbio.2022.05.006 35618044

[B45] Fields C. Levin M. (2020). How do living systems create meaning? Philosophies 5, 36. 10.3390/philosophies5040036

[B46] Fletcher A. (2021). Wonderworks: The 25 Most Powerful Innovations in the History of Literature, first Simon & Schuster Hardcover edition. New York, NY: Simon & Schuster.

[B47] Friedman M. (2008). Price Theory. Denver, CO: The Richest Man in Babylon.

[B48] Friston K. (2013). Life as we know it. J. R. Soc. Interface 10, 20130475. 10.1098/rsif.2013.0475 23825119 PMC3730701

[B49] Friston K. Frith C. (2015). A duet for one. Conscious. Cogn. 36, 390–405. 10.1016/j.concog.2014.12.003 25563935 PMC4553904

[B50] Friston K. Rigoli F. Ognibene D. Mathys C. Fitzgerald T. Pezzulo G.. (2015). Active inference and epistemic value. Cogn. Neurosci. 6, 187–214. 10.1080/17588928.2015.1020053 25689102

[B51] Friston K. J. Lin M. Frith C. D. Pezzulo G. Hobson J. A. Ondobaka S.. (2017). Active inference, curiosity and insight. Neural Comput. 29, 2633–2683. 10.1162/neco_a_00999 28777724

[B52] Friston K. J. Parr T. Yufik Y. Sajid N. Price C. J. Holmes E.. (2020). Generative models, linguistic communication and active inference. Neurosci. Biobehav. Rev. 118, 42–64. 10.1016/j.neubiorev.2020.07.005 32687883 PMC7758713

[B53] Fukuyama F. (1996). Trust: The Social Virtues and the Creation of Prosperity. A Free Press Paperbacks Book, 1. free press paperback edition. New York, NY: Free Press.

[B54] Greene J. D. (2013). Moral Tribes: Emotion, Reason, and the Gap between Us and Them. New York, NY: The Penguin Press.

[B55] Halvorson H. (2022). “The philosophy of science in Either-Or,” in Cambridge Critical Guide to Either-Or, eds R. Kemp, and W. Wietzke (Cambridge: Cambridge University Press), p. 19.

[B56] Hartung F.-M. Krohn C. Pirschtat M. (2019). Better than its reputation? Gossip and the reasons why we and individuals with “dark” personalities talk about others. Front. Psychol. 10, 1162. 10.3389/fpsyg.2019.01162 31191391 PMC6549470

[B57] Henrich J. (2016). The Secret of Our Success: How Culture Is Driving Human Evolution, Domesticating Our Species and Making Us Smarter. Princeton, NJ: Princeton University Press. 10.1515/9781400873296

[B58] Henrich J. P. (2020). The WEIRDest People in the World: How the West Became Psychologically Peculiar and Particularly Prosperous. New York, NY: Farrar, Straus and Giroux.

[B59] Henshilwood C. S. d'Errico F. van Niekerk K. L. Coquinot Y. Jacobs Z. Lauritzen S.-E.. (2011). A 100,000-year-old ochre-processing workshop at Blombos Cave, South Africa. Science 334, 219–222. 10.1126/science.1211535 21998386

[B60] Heyes C. M. (2018). Cognitive Gadgets: The Cultural Evolution of Thinking. Cambridge, MA: The Belknap Press of Harvard University Press. 10.4159/9780674985155

[B61] Heyes C. M. Frith C. D. (2014). The cultural evolution of mind reading. Science 344, 1243091. 10.1126/science.1243091 24948740

[B62] Hidalgo C. A. (2015). Why Information Grows: The Evolution of Order, from Atoms to Economies. New York, NY: Basic Books.

[B63] Hill K. R. Walker R. S. Božičević M. Eder J. Headland T. Hewlett B.. (2011). Co-residence patterns in Hunter-Gatherer societies show unique human social structure. Science 331, 1286–1289. 10.1126/science.1199071 21393537

[B64] Hofstadter D. R. Sander E. (2013). Surfaces and Essences: Analogy as the Fuel and Fire of Thinking. New York, NY: Basic Books.

[B65] Hogg M. A. (2000). Subjective uncertainty reduction through self-categorization: a motivational theory of social identity processes. Eur. Rev. Soc. Psychol. 11, 223–255. 10.1080/14792772043000040

[B66] Hohwy J. (2016). The self-evidencing brain: the self-evidencing brain. Noûs 50, 259–285. 10.1111/nous.12062

[B67] Isomura T. Parr T. Friston K. (2019). Bayesian filtering with multiple internal models: toward a theory of social intelligence. Neural Comput. 31, 2390–2431. 10.1162/neco_a_01239 31614100

[B68] Ivanhoe P. J. Van Norden B. W. (eds) (2005). Readings in Classical Chinese Philosophy, 2nd ed. Indianapolis, IN: Hackett Pub.

[B69] Jarvis E. D. (2006). Selection for and against vocal learning in birds and mammals. Ornithol. Sci. 5, 5–14. 10.2326/osj.5.5

[B70] Jehle G. A. Reny P. J. (2011). Advanced Microeconomic Theory, 3rd ed. Munich: Financial Times Prentice Hall.

[B71] Jung C. G. von Franz M.-L. Henderson J. L. Jacobi J. Jaffé A. (eds) (1968). Man and His Symbols. New York, NY: Laurel. Dell.

[B72] Kahneman D. (2003). Maps of bounded rationality: psychology for behavioral economics. Am. Econ. Rev. 93, 1449–1475. 10.1257/000282803322655392

[B73] Kay J. A. King M. A. (2020). Radical Uncertainty: Decision-Making beyond the Numbers, 1st ed. New York, NY: W. W. Norton &Company.

[B74] Keynes J. M. (2008). The General Theory of Employment, Interest, and Money. BN Pub.

[B75] Kinzler K. D. (2021). How You Say It: Why You Talk the Way You Do And What It Says about You. Boston, MA: MARINER BOOKS.

[B76] Kinzler K. D. Shutts K. DeJesus J. Spelke E. S. (2009). Accent trumps race in guiding children's social preferences. Soc. Cogn. 27, 623–634. 10.1521/soco.2009.27.4.623 21603154 PMC3096936

[B77] Knight F. H. (2014). Risk, Uncertainty and Profit.

[B78] Knop-Huelss K. Rieger D. Schneider F. M. (2020). Thinking about right and wrong: examining the effect of moral conflict on entertainment experiences, and knowledge. Media Psychol. 23, 625–650. 10.1080/15213269.2019.1623697

[B79] Lanillos P. Meo C. Pezzato C. Meera A. A. Baioumy M. Ohata W.. (2021). Active inference in robotics and artificial agents: survey and challenges. arXiv. Available online at: http://arxiv.org/abs/2112.01871

[B80] Leflot G. Onghena P. Colpin H. (2010). Teacher-child interactions: relations with children's self-concept in second grade. Infant Child Dev. 19, 385–405. 10.1002/icd.672

[B81] Levin M. (2019). The computational boundary of a “self”: developmental bioelectricity drives multicellularity and scale-free cognition. Front. Psychol. 10, 2688. 10.3389/fpsyg.2019.02688 31920779 PMC6923654

[B82] Levin M. Dennett D. (2020). Cognition All the Way Down. London: Aeon.

[B83] Liberman Z. Woodward A. L. Kinzler K. D. (2017). The origins of social categorization. Trends Cogn. Sci. 21, 556–568. 10.1016/j.tics.2017.04.004 28499741 PMC5605918

[B84] Lindley D. V. (1956). On a measure of the information provided by an experiment. Ann. Math. Stat. 27, 986–1005. 10.1214/aoms/1177728069

[B85] Lorenz K. (1974). On Aggression. A Harvest Book, HB 291. New York, NY: Harcourt Brace Jovanovich.

[B86] MacKay D. J. C. (1992). Information-based objective functions for active data selection. Neural Comput. 4, 590–604. 10.1162/neco.1992.4.4.590

[B87] MacKinnon N. J. Heise D. R. (2010). Social Reality and Human Subjectivity. New York, NY: Palgrave Macmillan US, 219–234. 10.1057/9780230108493_9

[B88] Masuda N. Fu F. (2015). Evolutionary models of in-group favoritism. F1000Prime Rep. 7, 27. 10.12703/P7-27 25926978 PMC4371377

[B89] McElreath R. Boyd R. Richerson P. J. (2003). Shared norms and the evolution of ethnic markers. Curr. Anthropol. 44, 122–130. 10.1086/345689

[B90] Moffett M. W. (2013). Human identity and the evolution of societies. Hum. Nat. 24, 219–267. 10.1007/s12110-013-9170-3 23813244

[B91] Moffett M. W. (2019). The Human Swarm: How Our Societies Arise, Thrive, and Fall. New York, NY: Basic Books.

[B92] Noble D. (2017). Dance to the Tune of Life: Biological Relativity. Cambridge: Cambridge University Press.

[B93] Palacios E. R. Razi A. Parr T. Kirchhoff M. Friston K. (2020). On Markov blankets and hierarchical self-organisation. J. Theor. Biol. 486, 110089. 10.1016/j.jtbi.2019.110089 31756340 PMC7284313

[B94] Parr T. Da Costa L. Friston K. (2020). Markov blankets, information geometry and stochastic thermodynamics. Philos. Trans. R. Soc. A Math. Phys. Eng. Sci. 378, 20190159. 10.1098/rsta.2019.0159 31865883 PMC6939234

[B95] Pfaff D. W. Wilson E. O. (2007). The Neuroscience of Fair Play: Why We (Usually) Follow the Golden Rule. New York, NY: Dana Press.

[B96] Ramstead M. J. D. Badcock P. B. Friston K. J. (2018). Answering Schrödinger's question: a free-energy formulation. Phys. Life Rev. 24, 1–16. 10.1016/j.plrev.2017.09.001 29029962 PMC5857288

[B97] Ramstead M. J. D. Sakthivadivel D. A. R. Heins C. Koudahl M. Millidge B. Da Costa K. J.. (2023). On Bayesian mechanics: a physics of and by beliefs. Interface Focus 13, 20220029. 10.1098/rsfs.2022.0029 37213925 PMC10198254

[B98] Ramstead M. J. D. Veissière S. P. L. Kirmayer L. J. (2016). Cultural affordances: scaffolding local worlds through shared intentionality and regimes of attention. Front. Psychol. 7, 1090. 10.3389/fpsyg.2016.01090 27507953 PMC4960915

[B99] Romer P. M. (1994). The origins of endogenous growth. J. Econ. Perspect. 8, 3–22. 10.1257/jep.8.1.310136763

[B100] Sakthivadivel D. A. R. (2022). Weak Markov blankets in high-dimensional, sparsely-coupled random dynamical systems. arXiv. 10.48550/ARXIV.2207.07620

[B101] Schein C. Gray K. (2018). The theory of dyadic morality: reinventing moral judgment by redefining harm. Pers. Soc. Psychol. Rev. 22, 32–70. 10.1177/1088868317698288 28504021

[B102] Schmidhuber J. (2006). Developmental robotics, optimal artificial curiosity, creativity, music, and the fine arts. Conn. Sci. 18, 173–187. 10.1080/09540090600768658

[B103] Searle J. (2010a). Searle: Philosophy of Language, Lecture 2.

[B104] Searle J. R. (2010b). Making the Social World: The Structure of Human Civilization. Oxford; New York, NY: Oxford University Press. 10.1093/acprof:osobl/9780195396171.001.0001

[B105] Seising R. (2012). “Warren weaver's “science and complexity” revisited,” in Soft Computing in Humanities and Social Sciences, Vol. 273, eds R. Seising, and V. Sanz González (Berlin: Springer), 55–87. 10.1007/978-3-642-24672-2_3

[B106] Simler K. Hanson R. (2018). The Elephant in the Brain: Hidden Motives in Everyday Life. New York, NY: Oxford University Press.

[B107] Simon H. A. (1992). Altruism and economics. East. Econ. J. 18, 73–83.

[B108] Slingerland E. G. (2014). Trying Not to Try: The Art and Science of Spontaneity, 1st ed. New York, NY: Crown Publishers.

[B109] Solms M. (2019). The hard problem of consciousness and the free energy principle. Front. Psychol. 9, 2714. 10.3389/fpsyg.2018.02714 30761057 PMC6363942

[B110] Solms M. (2022). Hidden Spring: A Journey to the Source of Consciousness. New York, NY: W. W. NORTON.

[B111] Strawson P. F. (2011). Individuals: An Essay in Descriptive Metaphysics. London: Routledge.

[B112] Tison R. Poirier P. (2021). Active inference and cooperative communication: an ecological alternative to the alignment view. Front. Psychol. 12, 708780. 10.3389/fpsyg.2021.708780 34456822 PMC8397515

[B113] Tomasello M. (2003). The Cultural Origins of Human Cognition. Cambridge, MA: Harvard Univ. Press.

[B114] Tomasello M. (2016). Cultural learning redux. Child Dev. 87, 643–653. 10.1111/cdev.12499 27189393

[B115] Vasil J. Badcock P. B. Constant A. Friston K. Ramstead M. J. D. (2020). A world unto itself: human communication as active inference. Front. Psychol. 11, 417. 10.3389/fpsyg.2020.00417 32269536 PMC7109408

[B116] Warneken F. Tomasello M. (2009). Varieties of altruism in children and chimpanzees. Trends Cogn. Sci. 13, 397–402. 10.1016/j.tics.2009.06.008 19716750

[B117] Whitehead A. N. (2010). Science and the Modern World. Number 1925 in Lowell Lectures. New York, NY: Free Press.

[B118] Wilson B. J. (2020). The Property Species: How “Mine” Makes Us Human, How “Yours” Makes Us Humane. New York, NY: Oxford University Press.

[B119] Wittgenstein L. Anscombe G. E. M. von Wright G. H. (1969). On Certainty. Number 1686 in Harper Torchbooks. New York, NY: Harper & Row.

